# Supplementary material for: Metaphorical framing of the COVID-19 pandemic in Pakistan: A corpus driven critical analysis of war metaphors in news media
Source: PLoS One. 2024 Oct 3;19(10):e0297115. doi: 10.1371/journal.pone.0297115 (PMC11449322; doi:10.1371/journal.pone.0297115)
Supplement: S1 Table — (PDF) [file pone.0297115.s001.pdf]

S1 Table: Size of PakNCovid-19 Corpus

Sr. No	Name of Newspaper	Month	No.of Editorials	Word Count
**1.**	Dawn	March	2	881
		April	4	1281
		May	7	2376
		June	3	1108
		July	5	1809
		**Size of Corpus**	**21**	**7,455**
**2.**	The Express Tribune	March	1	368
		April	3	921
		May	4	1288
		June	2	741
		July	3	1241
		**Size of Corpus**	**13**	**3,959**
**3.**	The News	March	1	421
		April	4	1589
		May	5	1934
		June	4	1367
		July	2	896
		**Size of Corpus**	**16**	**6,207**
	**Total**	**Size of Corpus**	**50**	**17,621**
