# Supplementary material for: Metaphorical framing of the COVID-19 pandemic in Pakistan: A corpus driven critical analysis of war metaphors in news media
Source: PLoS One. 2024 Oct 3;19(10):e0297115. doi: 10.1371/journal.pone.0297115 (PMC11449322; doi:10.1371/journal.pone.0297115)
Supplement: S2 Table — (PDF) [file pone.0297115.s002.pdf]

S2 Table. The steps involved in the Pragglejaz Method for Finding Metaphorically Used WORDS

**Step 1 – Decide about the boundaries of words**	**The excerpt 'doctors are frontline soldiers' does not literally mean that they are military persons**
**Step 2 – Establish the contextual meaning of the word being examined**	The contextual meaning of 'frontline soldiers' is to make doctors feel about their crucial duty to save others and be combatants
**Step 3 – Determine the basic meaning of the word**	The front line soldiers are the soldiers of opposing armies who are facing each other and where fighting is going on. [...] (Collins, 2023)
**Step 4 – Decide whether the basic meaning of the word is distinct from its contextual meaning**	The basic and contextual meanings of 'frontline soldiers' are distinct
**Step 5 – Decide whether there is some form of similarity between contextual meaning of the word that can be related to its basic meaning.**	During Covid-19, the doctors have the major responsibility of saving lives, treating infected people, and disseminating precautionary measures, and are known to be saviors of country. Similarly, soldiers save their country in a war
