# Supplementary material for: Metaphorical framing of the COVID-19 pandemic in Pakistan: A corpus driven critical analysis of war metaphors in news media
Source: PLoS One. 2024 Oct 3;19(10):e0297115. doi: 10.1371/journal.pone.0297115 (PMC11449322; doi:10.1371/journal.pone.0297115)
Supplement: S3 Table — (PDF) [file pone.0297115.s003.pdf]

S3 Table. War metaphors of Covid-19 discourse in Pakistan

Source Domain	Target Domain	Metaphor Formula	Explanation of Conceptual Metaphors
**War against Covid-19**	Control, Victory	WAR AGAINST COVID-19	The pandemic is framed as a war, emphasizing the need for control and achieving victory.
**Covid-19 as an enemy**	Confrontation	COVID-19 AS AN ENEMY	Covid-19 is metaphorically portrayed as an adversary that needs to be confronted and overcome.
**Battle against Covid-19**	Challenge, Victory	BATTLE AGAINST COVID-19	The response to Covid-19 is likened to a battle involving challenges and the pursuit of victory.
**Doctors as soldiers**	Dedication, Defense	DOCTORS AS SOLDIERS	Doctors are depicted as dedicated soldiers on the frontlines, defending against the pandemic.
**Fight against Covid-19**	Opposition, Triumph	FIGHT AGAINST COVID-19	The response to the pandemic is framed as a fight against opposition, with the goal of achieving triumph.
**Victory over Covid-19**	Achievement, Success	VICTORY OVER COVID-19	The objective is to achieve victory over the pandemic, signifying success and overcoming challenges.
