# Supplementary figures and images for: Metaphorical framing of the COVID-19 pandemic in Pakistan: A corpus driven critical analysis of war metaphors in news media
Source: PLoS One. 2024 Oct 3;19(10):e0297115. doi: 10.1371/journal.pone.0297115 (PMC11449322; doi:10.1371/journal.pone.0297115)

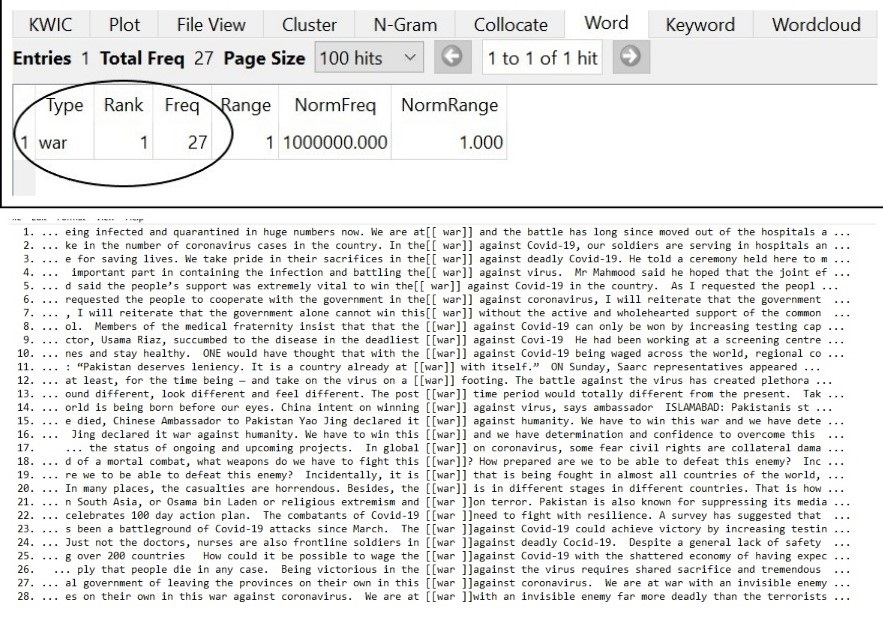

Supplement: S1 Fig — (TIF) [file pone.0297115.s004.tif]

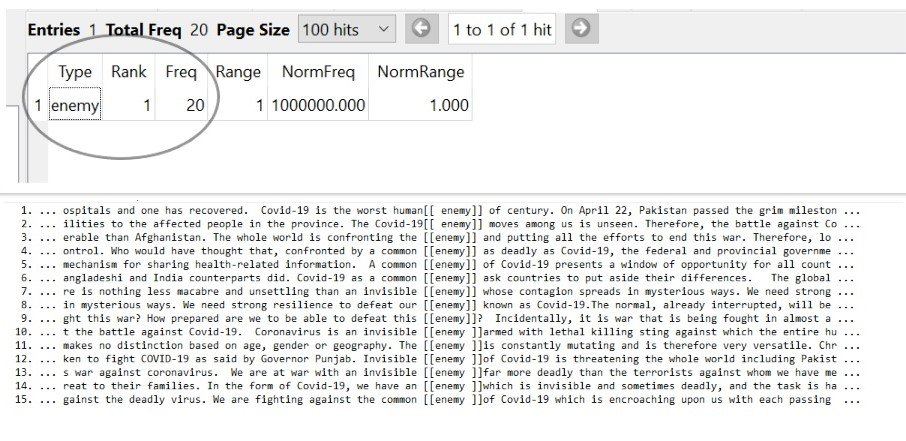

Supplement: S2 Fig — (TIF) [file pone.0297115.s005.tif]

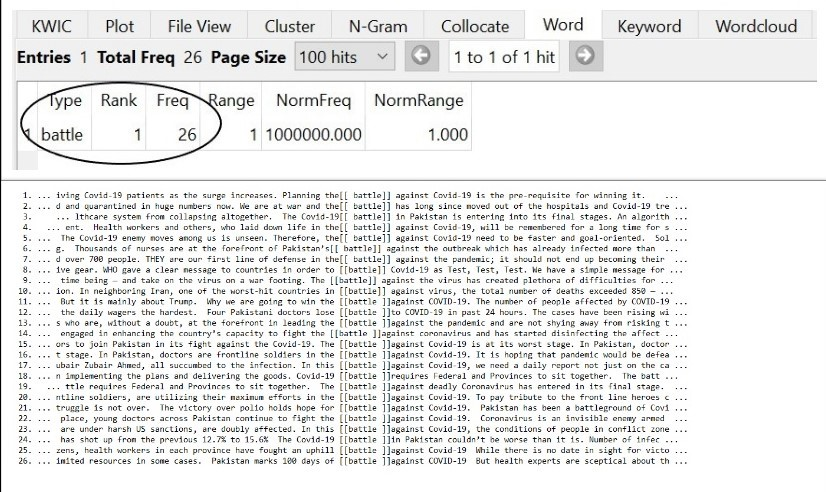

Supplement: S3 Fig — (TIF) [file pone.0297115.s006.tif]

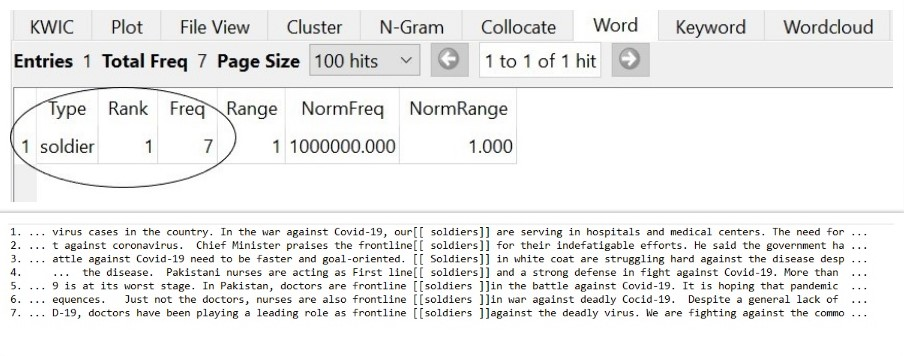

Supplement: S4 Fig — (TIF) [file pone.0297115.s007.tif]

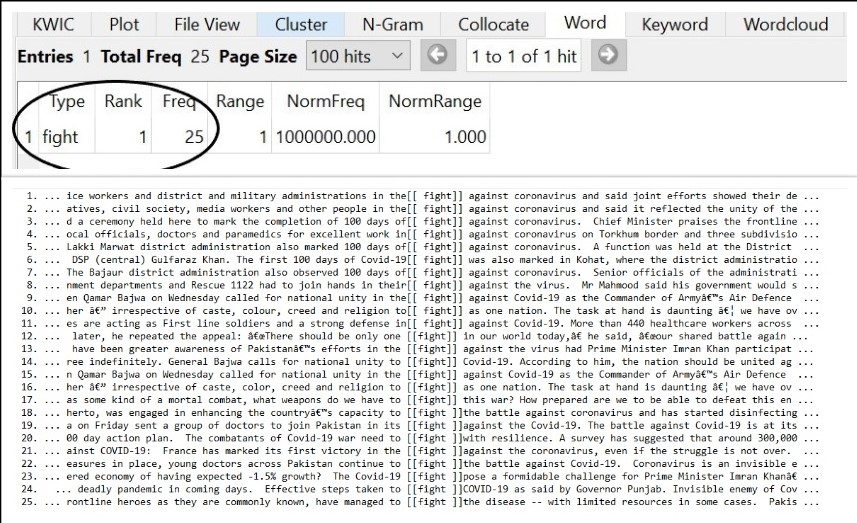

Supplement: S5 Fig — (TIF) [file pone.0297115.s008.tif]

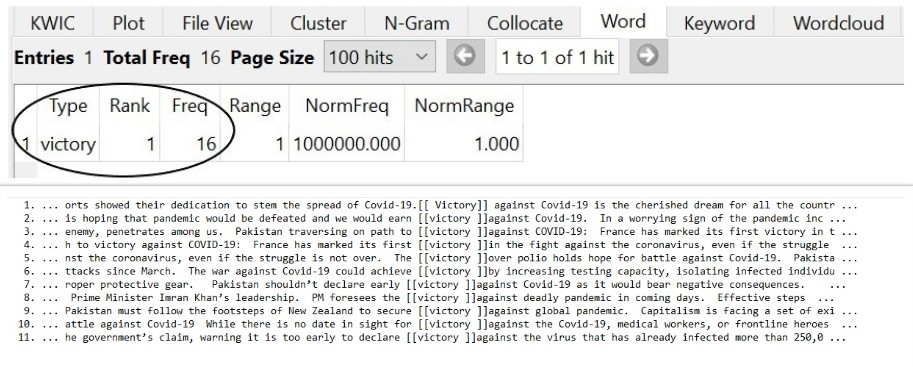

Supplement: S6 Fig — (TIF) [file pone.0297115.s009.tif]
