# Supplementary material for: Metaphorical framing of the COVID-19 pandemic in Pakistan: A corpus driven critical analysis of war metaphors in news media
Source: PLoS One. 2024 Oct 3;19(10):e0297115. doi: 10.1371/journal.pone.0297115 (PMC11449322; doi:10.1371/journal.pone.0297115)
Supplement: Attachment — Submitted filename: respose to Reviewers.docx [file pone.0297115.s011.docx]

PLOS One

San Francisco, California, US

Dear editors,

We thank the reviewers for their generous comments on the manuscript and have have edited the manuscript to address their concerns.

In particular, we have added a table containing quantitative data and also added the frequency tables of each key word. Furthermore, bibliography has been updated and relevant researches for war metaphor in relation to Covid-19 have been included. Moreover, obsolete references have been omitted as suggested by the reviewers. In the end, the results have immersed by the similar analyses conducted by other scholars.

We believe that the manuscript is now suitable for publication in PLOS One.

Regards,

Arooj Rana,

Lecturer, Quaid-I-Azam University Islamabad

On behalf of all authors.
