# Supplementary material for: Metaphorical framing of the COVID-19 pandemic in Pakistan: A corpus driven critical analysis of war metaphors in news media
Source: PLoS One. 2024 Oct 3;19(10):e0297115. doi: 10.1371/journal.pone.0297115 (PMC11449322; doi:10.1371/journal.pone.0297115)
Supplement: Attachment — Submitted filename: Response to Reviewers.docx [file pone.0297115.s012.docx]

PLOS One

San Francisco, California, US

Dear editors,

We thank the reviewers for their generous comments on the manuscript and have edited the manuscript to address their concerns.

In particular, we have added convincing arguments related to recent developments in metaphors and extended conceptual metaphor theory (Kövecses, Z. 2016; Kövecses, Z. 2020; Kövecses, Zoltán. 2021). We have also added recent studies on Covid-19 conceptual metaphors in the study and have drawn cross-linguistic and cultural implications. We have updated the title of the manuscript as well. The section on 'Traditional approaches to metaphor" has been deleted and literature review has been revised by adding more convincing and recent references of studies as per reviewers’ suggestions. Corpus-driven approach has been mentioned in the study and metaphorical expressions were identified using the well-established Pragglejaz Method for Finding Metaphorically Used Words (MIP) (the reference table has been added in Appendix 1). The discussion section is added with relevant studies and a more in-depth discussion of the implication are provided. The structure of the paper is redesigned as follows: abstract, introduction, CMT and medical discourse, method and material, data analysis, conclusion. The literature of the paper is rewritten by adding metaphor in medical discourses, and other papers related to politics and etc., have been removed. Moreover, justification for choosing war metaphors have also been added under a separate heading. Metaphor formula for all the source domains have also been added in a tabular form.

We believe that the manuscript is now suitable for publication in PLOS One.

Regards,

Arooj Rana,

Lecturer, Quaid-I-Azam University Islamabad

On behalf of all authors.
