# Supplementary material for: Metaphorical framing of the COVID-19 pandemic in Pakistan: A corpus driven critical analysis of war metaphors in news media
Source: PLoS One. 2024 Oct 3;19(10):e0297115. doi: 10.1371/journal.pone.0297115 (PMC11449322; doi:10.1371/journal.pone.0297115)
Supplement: Attachment — Submitted filename: Response to Reviewers.docx [file pone.0297115.s014.docx]

PLOS One

San Francisco, California, US

Dear editors,

We thank the Academic Editor for the generous comments on the manuscript and have edited the manuscript to address the concerns.

In particular, we have edited the numbers of citations and bibliography. Moreover, the Table Numbers have been carefully edited and a similar patter have been followed for citing all the tables. Table 2 have also been moved inside the text for readers’ facilitation. While addressing the third concern of researcher agreement, the method and index of agreement have been added in the research paper. Verb tenses have also been edited as per the editor’s demands. The concordance Index section is modified as suggested by the reviewers on Dec. 4 2023.

We believe that the manuscript is now suitable for publication in PLOS One.

Regards,

Arooj Rana,

Lecturer, Quaid-i-Azam University Islamabad

On behalf of all authors.

28^th^ October, 2023.
